# SVJedi-graph: improving the genotyping of close and overlapping structural variants with long reads using a variation graph

**DOI:** 10.1093/bioinformatics/btad237

**Published:** 2023-06-30

**Authors:** Sandra Romain, Claire Lemaitre

**Affiliations:** Univ Rennes, Inria, CNRS, IRISA, Rennes F-35000, France; Univ Rennes, Inria, CNRS, IRISA, Rennes F-35000, France

## Abstract

**Motivation:**

Structural variation (SV) is a class of genetic diversity whose importance is increasingly revealed by genome resequencing, especially with long-read technologies. One crucial problem when analyzing and comparing SVs in several individuals is their accurate genotyping, that is determining whether a described SV is present or absent in one sequenced individual, and if present, in how many copies. There are only a few methods dedicated to SV genotyping with long-read data, and all either suffer of a bias toward the reference allele by not representing equally all alleles, or have difficulties genotyping close or overlapping SVs due to a linear representation of the alleles.

**Results:**

We present SVJedi-graph, a novel method for SV genotyping that relies on a variation graph to represent in a single data structure all alleles of a set of SVs. The long reads are mapped on the variation graph and the resulting alignments that cover allele-specific edges in the graph are used to estimate the most likely genotype for each SV. Running SVJedi-graph on simulated sets of close and overlapping deletions showed that this graph model prevents the bias toward the reference alleles and allows maintaining high genotyping accuracy whatever the SV proximity, contrary to other state of the art genotypers. On the human gold standard HG002 dataset, SVJedi-graph obtained the best performances, genotyping 99.5% of the high confidence SV callset with an accuracy of 95% in less than 30 min.

**Availability and implementation:**

SVJedi-graph is distributed under an AGPL license and available on GitHub at https://github.com/SandraLouise/SVJedi-graph and as a BioConda package.

## 1 Introduction

Structural variants (SVs) are genomic rearrangements of at least 50 bp that differ between the genomes of individuals belonging to the same species. This definition encompasses a wide range of variations in terms of size and type. The most frequent types are deletions and insertions, but there are also balanced SVs such as inversions and translocations. Although SVs are less frequent in numbers than punctual variations, they often involve more base pairs in the genomes and have long been shown to be involved in phenotypic variability, species adaptation and evolution, and in many diseases and disorders (Weischenfeldt et al. 2013; Mahmoud et al. 2019).

With the democratization of long-read sequencing technologies, there has been an increasing number of studies focusing on the characterization and analysis of this type of genetic variation on a genome-wide scale in various organisms. Indeed, because of their large size and frequent localization in repeated regions, SVs were very challenging variants to identify with short reads (Mahmoud et al. 2019; Delage et al. 2020). Long reads have really changed the game in this field, allowing their reliable and accurate detection in resequencing genome data (Chaisson et al. 2019; Zook et al. 2020). In particular, in recent years, numerous studies have been conducted at the population level with large sample sizes, revealing associations between SVs and phenotypes of interest or their involvement in changes in gene expression in various organisms, such as, for example, in plants (Alonge et al. 2020), in yeasts (O’Donnell et al. 2022) and of course in human populations (Beyter et al. 2021; Porubsky et al. 2022), to cite only a few of them.

In most of these studies, the input of the analyses is typically a matrix with variants in lines and samples or individuals in columns (or *vice versa*) containing in each cell the genotype or number of each allele of the given variant in the given individual. To obtain such a matrix, the commonly accepted approach is composed of two steps: the first one consists in obtaining a most comprehensive and nonredundant set of SVs. This is achieved by using SV discovery tools on all or a subset of the samples to identify all structural variants in samples compared to a reference genome. The obtained call sets are then merged to obtain a nonredundant set of SVs which defines the lines of the matrix. Then, the second step is the genotyping and aims at filling the matrix with genotypes. Genotyping one variant in an individual consists in counting how many reads from this individual support each described allele of the given variant. Based on these read counts, a genotype is derived, typically homozygous for the reference or alternative allele or heterozygous for bi-allelic variants in a diploid individual. In such a genotyping step, all samples are thus evaluated through the same SV call set to obtain comparable values.

Genotyping and discovery are therefore two distinct tasks that necessitate different methods and we have witnessed a strong increase in the number of tools developed in recent years dedicated purely to the genotyping problem. While genotyping appears as a simpler problem than discovery, since variants are already known and the whole reference genome does not need to be blindly investigated, there are some issues that deserve special attention. The first issue is named the reference bias. It is well known that the more similar two sequences are, the easier it is to align them and this is emphasized in the context of structural dissimilarity and with reads containing many sequencing errors. Therefore, when mapping reads only to the reference genome, one may favor the reference allele. As the different alleles are well defined in the genotyping problem, the reference bias should be avoidable, by mapping the reads on both reference and alternative alleles in an equal manner. This can be achieved by generating allele-specific subsequences and mapping the reads only on these sequences. Typically, these sequences are centered on the SV breakpoints and include neighboring sequences of size depending on the average read size.

The second issue concerns closely located or overlapping SVs for which representing the sequences of the different alleles is not so trivial. For a given SV, the neighboring sequences are not uniquely defined as they depend on the allele states of neighboring SVs. Intuitively, moving from a linear to a graph-based representation solves this issue. In a variation graph, each node is a sequence, edges represent adjacencies between sequences observed in an allele and each combination of alleles is represented by a path in this graph. As a matter of fact, numerous implementations for building variation graphs or also named pangenome graphs, analyzing such graphs or mapping reads on them are now available and commonly used (see Paten et al. 2017; Garrison et al. 2018; Li et al. 2020, Rautiainen and Marschall 2020; Guarracino et al. 2022 to cite only a few). Importantly, they have been shown to improve read mapping and small variant genotyping (Eggertsson et al. 2017; Garrison et al. 2018). As concerns SVs, the last generation of SV genotypers for short Illumina reads, Paragraph (Chen et al. 2019), graphTyper2 (Eggertsson et al. 2019), the latest Giraffe (Sirén et al. 2021), and PanGenie (Ebler et al. 2022), are all based on such sequence graphs.

As genome sequencing is more and more achieved with long-read technologies, even for population scale studies (Coster et al. 2021), and because the large size of the reads has an undeniable benefit on the quality of SV analyses (for genotyping as well as for discovery) (Mahmoud et al. 2019, Duan et al. 2022), a few tools dedicated to SV genotyping with long-read data have been proposed these last years, namely VaPoR (Zhao et al. 2017), SVJedi (Lecompte et al. 2020), and LRcaller (Beyter et al. 2021). However, none of them uses a graph representation of the variants. All three tools explicitly represent the allelic sequences, but as linear sequences, and map the reads on both reference and alternative allele sequences. However, only SVJedi strictly avoids the reference bias by mapping all the reads on all allelic sequences, whereas VaPoR and LRcaller perform first a selection of the reads to be mapped on alleles based on a whole reference genome alignment given as input. Additionally, Sniffles (Sedlazeck et al. 2018) and CuteSV (Jiang et al. 2020), which are discovery tools, also provide a genotyping mode as an option, but the methods implemented for these optional modes have not been described in any publication. As these tools require a mapping on the reference genome as input, we can hypothesize that they mainly rely on the split-read signatures used also in their discovery mode and may thus be subject to the reference bias.

We present here the first SV genotyper for long-read data that is based on a variation graph. By avoiding the mapping on the reference genome only and using a variation graph representing the whole reference genome complemented by all described alternative alleles given in the input SV call set, our method is not reference-biased and improves the genotyping of distant, as well as closely located and overlapping SVs.

## 2 Materials and methods

Our method relies on the representation of structural variants with a variation graph, which is then used as “reference” to map the long reads on.

It takes as input the set of SVs to genotype in VCF format, the sequence of the reference genome in FASTA format, and the long reads from which the SV genotypes will be estimated in FASTQ or FASTA format (compressed or not). The main output is a VCF file, corresponding to the input VCF file with an additional column containing the predicted genotypes of the SVs. It also outputs the variation graph representing the whole genome and alternative alleles of the input SVs in GFA format.

**Figure 1.**
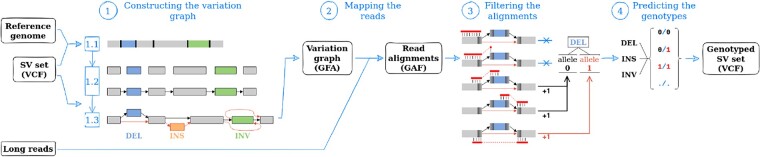
Illustration of the four steps of SVJedi-graph. The method takes three files as input: the sequence of the reference genome, the VCF describing the SVs to genotype, and the long reads to genotype the SVs from. The first step is the construction of the variation graph, the second step is the mapping of the long reads on the variation graph with minigraph (producing the GAF alignment file), the third step is the filtering of the reads, and the final fourth step is the genotype prediction. Two files are output, with the main one being the genotyped version of the input VCF, and the other one being the GFA containing the variation graph.

Our method is composed of four steps, illustrated in Fig. 1. First, we build the variation graph from the reference genome and the SV set. We then use an external tool, minigraph (Li et al. 2020), to map the long reads on the graph we produced. The alignment results are filtered to identify genotype-informative reads, which are stored by covered SV and supported allele. Finally, the read counts are normalized and we attribute the genotype with the maximum likelihood to each SV of the input set.

### 2.1 Constructing the variation graph

A variation graph is a directed graph whose nodes are labeled with nonoverlapping genomic sequences. Edges represent sequence adjacencies observed in a genome or allelic sequence. A path in the graph represents a possible haplotype in a genome.

In our method, we construct a variation graph from the sequence of the reference genome and a set of SVs characterized by their type, their breakpoint positions on the reference genome, and their sequence in the case of insertions. The first step is to list and sort all breakpoint positions for each chromosome of the reference genome, then use them in the second step to fragment the reference sequence of the chromosome into reference nodes. Reference edges are added between each pair of successive reference nodes, forming the path of the reference genome in the graph.

The third step is to add additional edges for each SV described in the input VCF according to its type to form the path of the alternative allele. We call such edges alternative edges. In the case of insertions, an alternative node is also added, labeled with the sequence of the insertion. Thus, in our variation graph, all edges represent breakpoints of the input SVs, that is sequence adjacencies that are specific to one of the alleles.

The resulting variation graph is output in the GFA format.

In our graph, we currently can represent deletions, insertions, inversions, and intra-chromosomal translocations. The first step of the construction allows for the representation of overlapping SVs.

### 2.2 Mapping the reads on the graph

The long reads are mapped on the constructed variation graph with minigraph (v0.19) (Li et al. 2020), with the “-x lr” argument for aligning long reads and without base-level alignment to increase speed since only the read position on the graph is needed in our method to predict the SV genotype. Minigraph outputs the alignments’ results in the GAF format, which is a variation of the PAF format adapted to sequence graphs.

We have also tested another mapper, GraphAligner (Rautiainen and Marschall 2020) and the base-level alignment mode of minigraph. We chose minigraph without base-level alignment which gave the best results.

### 2.3 Selecting the informative reads

In our method, we consider that a read aligning on at least one breakpoint sequence of an SV gives information on that SV’s genotype, since breakpoints are sequence adjacencies specific to one or the other SV allele. Each SV has one or two breakpoints for each of its alleles depending on its type. For example, deletions have two breakpoints for their reference allele, that we will call reference breakpoints, and one breakpoint for their alternative allele, that we will call alternative breakpoint. Inversions have two reference breakpoints and two alternative breakpoints. Each breakpoint is represented by a distinct edge in the variation graph. For each alignment output by minigraph, we first verify that the read aligns on at least two nodes, meaning that it overlaps at least one of the breakpoints in the graph. Then, we list all SVs that have at least one breakpoint included in the span of the read alignment on the graph. For each of the listed SVs, we determine which allele is covered by the alignment and increment by one the support value for this SV’s allele for each allele breakpoint that the read alignment covers. A read is considered as covering a breakpoint if the alignment overlaps at least *d*_over_ base pair from each side of the breakpoint. We fixed the default value of *d*_over_ at 100 bp.

Each time a read is counted as allele support for an SV, the support count for this SV’s allele is incremented by one in a dictionary containing the SVs as keys. If a read covers both breakpoints of an SV allele, it counts as two read supports.

### 2.4 Predicting the SV genotypes

Once all the alignments produced by minigraph have been processed, the genotype of each SV is estimated using the read support counts for its alleles. First, for SV types with an unbalanced number of breakpoints between alleles (deletions and insertions), the support count is normalized for each allele by the allele’s breakpoint number (e.g. for a deletion, the reference allele count is divided by two). Then, the normalized allele support counts are used to compute the likelihood for each possible genotype in a diploid individual (homozygous for reference 0/0, heterozygous 0/1, or homozygous for alternative 1/1). We use the same likelihood formula as in SVJedi and CuteSV (Lecompte et al. 2020; Jiang et al. 2020), which is described in Nielsen et al. (2011). Basically, the likelihoods of the three possible genotypes given the observed normalized read counts (*c*_0_ and *c*_1_ for reference and alternative alleles, respectively) are computed based on a simple binomial model: where *p*_e_ is the probability that a read maps to a given allele erroneously, assuming it is constant, and independent between all observations. *p*_e_ was fixed to 5 × 10^−5^, after empirical experiments. The genotype with the largest likelihood is assigned and all three likelihoods are also output (−log10 transformed) as additional information in the VCF file.


(1) $$L(0/0)={\left(1-p_{e}\right)}^{c_{0}}\times p_{e}^{c_{1}}\times C_{c_{0}+c_{1}}^{c_{0}}$$

(2) $$L(1/1)=p_{e}^{c_{0}}\times {\left(1-p_{e}\right)}^{c_{1}}\times C_{c_{0}+c_{1}}^{c_{0}}$$

(3) $$L(0/1)={\left(\frac{1}{2}\right)}^{c_{0}+c_{1}}\times C_{c_{0}+c_{1}}^{c_{0}}$$

Finally, we report the genotype of an SV only if it is supported by a minimal amount of supporting reads (sum of allele counts after normalization), otherwise a missing genotype (“./.”) is reported. This is governed by a user-defined parameter, whose default value is set to 3.

### 2.5 Implementation

The presented method is implemented in Python under the name SVJedi-graph (v1.1.1) and is available on github (https://github.com/SandraLouise/SVJedi-graph) and as a conda package (https://anaconda.org/bioconda/svjedi-graph). Currently, SVJedi-graph can genotype five types of SVs: deletions, insertions, duplications, inversions, and intra-chromosomal translocations. Insertions need to be sequence-resolved with the full inserted sequence characterized and reported in the ALT field of the VCF file. As duplications are a special case of insertions, SVJedi-graph supports also duplications, as long as their duplicated sequence is characterized and reported similarly to insertions.

### 2.6 Simulating close and overlapping SV datasets

In order to evaluate our method’s genotyping performances on closely located or overlapping SVs, we simulated twelve deletion datasets with varying distance ranges between deletions on the human chromosome 1 (assembly GRCh37.p13). All those datasets shared the same 995 deletions selected from the dbVar database (Phan et al. 2017), ranging from 50 bp to 10 kb in size and distant of at least 10 kb from each other. These deletions were equally distributed over the three possible genotypes (0/0, 0/1, 1/1), resulting in two synthetic haplotype sequences of chromosome 1. These haplotype sequences were used to simulate a single long-read sequencing dataset using simLoRD (Stöcker et al. 2016), with a PacBio error profile and error rate of 16% and at a sequencing depth of 30×.

Then, we generated 12 different variant sets (VCF files) by adding to each of those 995 “initial” deletions one simulated deletion at different distance or overlapping ranges from its companion deletion. The size of these additional deletions ranged from 50 bp to 2 kb, and they were all recorded as homozygous reference genotype (0/0) in the variant file, meaning that these additional deletions are not present in the simulated sequenced individual. Therefore, the same set of simulated reads can be used to genotype the different deletion sets. Six of the deletion sets correspond to nonoverlapping deletions, with random distance of: (i) 5–10 kb, (ii) 1–5 kb, (iii) 500–1 kb, (iv) 100–500 bp, (v) 50–100 bp, and (vi) 0–50 bp. They contain each 1990 deletions with a median size around 1 kb. The other six sets simulated overlapping deletions, with random overlapping of: (i) 0–50 bp, (ii) 50–100 bp, (iii) 100–200 bp, (iv) 200–300 bp, (v) 300–400 bp, and (vi) 400–500 bp. The size of the sets varies depending on the overlap size range, since only the deletions larger than the minimal overlap bound were kept. These overlapping deletion sets contain between 1382 (400–500 bp overlaps) and 1990 deletions (0–50 bp overlaps). Accordingly, deletions are larger in the sets with the largest overlap sizes (the median deletion size ranges from 1 to 1.3 kb). All simulated datasets are available for download (see Supplementary Material).

### 2.7 Evaluation and comparison to state of the art long-read genotypers

We evaluated and compared our method to other genotypers on its genotyping quality and computing performances. To evaluate the genotyping quality, we used two metrics: the genotyping accuracy and the genotyping rate.

We define the genotyping rate as the percentage of input SVs for which the tool was able to attribute a genotype. It was calculated using Equation (4), where TP is the number of SVs for which the predicted genotype corresponds to the true genotype, FP is the number of SVs for which the predicted genotype differs from the true genotype, and FN is the number of SVs that could not be attributed a genotype. We define the genotyping accuracy as the percentage of genotyped SVs that were attributed their true genotype, calculated with Equation (5).


(4) $$\mathrm{Genotyping}\;\mathrm{rate}=\frac{\mathrm{TP}+\mathrm{FP}}{\mathrm{TP}+\mathrm{FP}+\mathrm{FN}}\times 100$$

(5) $$\mathrm{Genotyping}\;\mathrm{accuracy}=\frac{\mathrm{TP}}{\mathrm{TP}+\mathrm{FP}}\times 100.$$

We compared our method to four state of the art long-read SV genotypers, namely SVJedi (Lecompte et al. 2020) (v1.1.6), cuteSV (Jiang et al. 2020) (v1.0.13), Sniffles2 (Sedlazeck et al. 2018) (v2.0.6), and LRcaller (Beyter et al. 2021) (v1.0). SVJedi and LRcaller are tools dedicated to SV genotyping, while cuteSV and Sniffles2 are primarily SV callers. CuteSV and Sniffles2 were run with their “force call” option to genotype a given set of SVs, bypassing the SV discovery steps. For each comparison, all tools were run with the same variant file as input. SVJedi performs the read mapping internally using minimap2, while cuteSV, Sniffles2, and LRcaller take as input the results of the read mapping done externally. SVJedi was run on the ONT reads with the parameter “-d ont,” and on both PacBio datasets with the default parameter “-d pb.” As SVJedi uses minimap2 (v2.17), we also used minimap2 (Li 2018) (v2.17) to map the reads on the reference genome, as input to cuteSV, Sniffles2, and LRcaller. Minimap2 was run on the PacBio CLR reads, PacBio HiFi reads, and ONT reads with the parameters presets “map-pb,” “asm20,” and “map-ont,” respectively. LRcaller has five methods to genotype SVs, we used the joint method as done in the benchmark paper for SV genotyping methods (Duan et al. 2022) with the argument “–gtm joint.” All tools but Sniffles2 were run using 20 CPU threads. Command lines used to run the tools are given in Supplementary Material.

## 3 Results

### 3.1 Impact of SV proximity in simulated datasets

In order to evaluate the benefits of using a variation graph to genotype SVs, and in particular close and overlapping SVs, we first applied our method to several simulated datasets of deletions in the human chromosome 1, in which we controlled the distance or overlap size of consecutive pairs of deletions (see Section 2).

**Figure 2.**
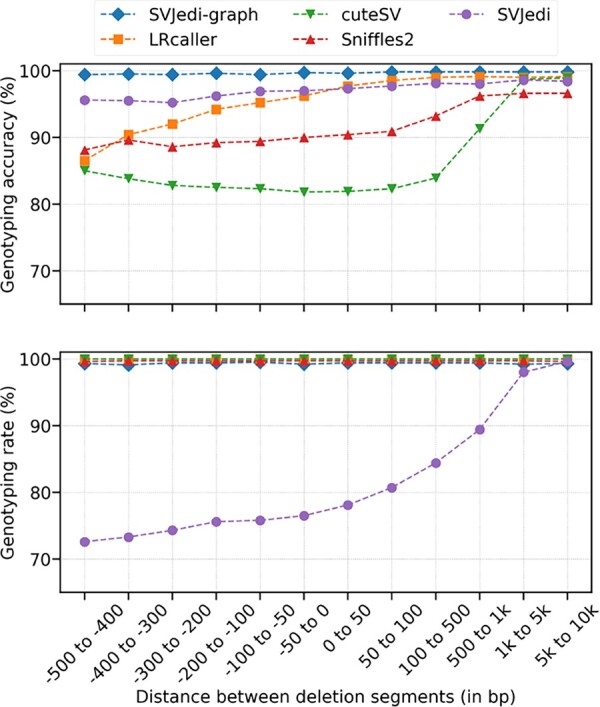
Genotyping performances of long-read SV genotypers on the 12 simulated deletion datasets on human chromosome 1, with varying distances between pairs of consecutive deletions. The *X* axis represents the different simulated datasets ordered by increasing distance between pairs of consecutive deletion segments. Negative *X* values correspond to datasets with overlapping deletions.

Figure 2 shows the performance metrics of SVJedi-graph and the other compared genotypers as a function of the distance between pairs of consecutive deletion segments. We observe that the distance between deletions and the fact that some deletions overlap each other does not impact SVJedi-graph performances. It maintains very high accuracy and rate (above 99%) whatever the distance between the simulated deletions and even for overlapping deletions.

On the contrary, all the other tested genotypers show decreasing genotyping qualities when the deletions are closer to one another. CuteSV and Sniffles genotyping accuracy starts decreasing as soon as deletions are <1000 bp apart, falling ∼80% and 90%, respectively, for overlapping or adjacent deletions. The drop in accuracy is smaller for LRcaller, which maintains its accuracy above 97% for even very close deletions, but falls below 90% for overlapping deletions with the largest overlaps. On the other hand, SVJedi maintains its high accuracy but its genotyping rate decreases regularly with the deletion proximity. It is not able to assign a genotype to more than 20% of the deletions that are <50 bp apart.

### 3.2 Impact of breakpoint position precision in simulated datasets

In practice, real SV call sets may not be defined at the base pair resolution and can contain breakpoint positions shifted from the real positions. In order to evaluate to what extend this imprecision in breakpoint definition may impact the genotyping performances, we applied the genotypers on the previous simulated long-read dataset but with imprecise input VCF files, where the positions of the 995 deletions used to simulate the reads were shifted. Both breakpoints of the deletions were shifted by a fixed distance in the same direction to preserve the deletion size and we retained only those deletions where the deleted segment overlapped by at least 50% with the deletion from which it was moved.

**Figure 3.**
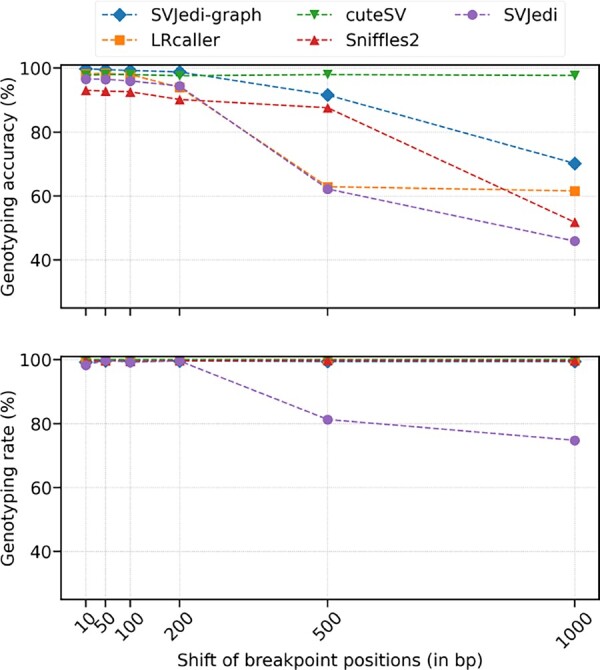
Genotyping performances of long-read SV genotypers on simulated deletion datasets on human chromosome 1 with varying levels of imprecision in the breakpoint definitions. The *X* axis represents the distance in base pairs between the deletions breakpoints as simulated in the input read dataset and their corresponding shifted breakpoints given in the different input VCF files.

Figure 3 shows the performance metrics of SVJedi-graph and the other compared genotypers when increasing the breakpoint shift from 10 to 1000 bp. Except for cuteSV which remarkably seems not to be impacted by breakpoints shifts, all other genotypers show decreasing genotyping accuracies when the imprecision increases. SVJedi-graph still maintains a high accuracy as long as the imprecision is smaller than 200 bp (98.8% for 200 bp breakpoint shifts).

### 3.3 Results on real human benchmark datasets

To assess genotyping accuracy on real data, one needs a comprehensive set of well characterized SVs with their genotype well ascertained in at least one individual. The consortium Genome in a Bottle (GIAB), thanks to massive data production and manual efforts, produced such a dataset dedicated to SV tools benchmarking on the human individual HG002, son of the so-called *Ashkenazi trio* (Zook et al. 2020). This highly curated set, referred as High confidence, contains 5464 deletions and 7281 insertions of at least 50 bp which are distant from one another from at least 1 kb, whose genotypes are heterozygous or homozygous for the alternative allele in HG002 (Tier 1 set v.0.6 with the tag “PASS” in the VCF FILTER field) We applied SVJedi-graph on this set with three long-read datasets from the HG002 individual obtained with different sequencing technologies, namely PacBio CLR, PacBio HiFi and Nanopore (ONT), and provided by GIAB (see Supplementary Material for download links).

With the 30× CLR PacBio reads, SVJedi-graph was able to genotype 99% of the SVs with a genotyping accuracy of 94.6% (Tables 1 and 2). This accuracy is slightly better for deletions than for insertions for this set (1.3 point %), as well as the genotyping rate (1.6 point %). Table 1 shows a contingency table of the obtained genotypes compared with expected ones for deletions and insertions. Most genotyping errors happen on alternative homozygous (1/1) deletions or insertions that end up predicted as heterogygous (0/1). Out of the errors made on alternative homozygous variants, 99% and 98% were wrongly predicted heterozygous deletions and insertions, accounting for 56% and 77% of all genotyping errors for each of these SV types, respectively. For heterozygous deletions, the errors are well balanced between wrongly genotyped reference homozygous and alternative homozygous, especially for insertions (50% for each of the two possible wrong genotypes). For heterozygous deletions, 69% of the errors were alternative homozygous genotype predictions.

**Table 1.** Contingency tables of SVJedi-graph genotyping results on the real 30× PacBio dataset of human individual HG002 with respect to the high confidence GIAB call set.^a^

Deletions					
		SVJedi-graph predictions			
		0/0	0/1	1/1	./.
**GIAB**	0/1	34	3322	75	2
	1/1	2	142	1884	3

Insertions					
		SVJedi-graph predictions			
		0/0	0/1	1/1	./.
**GIAB**	0/1	47	3397	46	15
	1/1	6	324	3339	107

a Results for the 5464 deletions (top) and 7281 insertions (bottom) are indicated in two separated tables, where columns indicate SVJedi-graph genotypes and rows GIAB ones. Gray labeled boxes, in the diagonal, give the amount of variants correctly genotyped by SVJedi-graph. The number of genotypes that SVJedi-graph fails to assess is indicated by the “./.” column.

**Table 2.** Genotyping accuracy and rate of SVJedi-graph and state of the art genotyping tools on the deletions and insertions of the *High confidence* HG002 SV set with the 30× CLR PacBio read dataset.

	Global		Deletions		Insertions	
Tool	Accuracy (%)	Rate (%)	Accuracy (%)	Rate (%)	Accuracy (%)	Rate (%)
SVJedi-graph	94.6	99.0	95.4	99.9	94.1	98.3
cuteSV	88.7	100	90.8	100	87.1	100
LRcaller	83.6	100	89.3	100	79.3	100
Sniffles2	85.4	99.5	87.9	99.9	83.6	99.1
SVJedi	92.2	90.2	91.7	85.8	92.5	93.6

The four other tested genotyping tools present a lower accuracy than SVJedi-graph on the global SV set (92.2%, 88.7%, 85.4%, and 83.6% for SVJedi, cuteSV, Sniffles2, and LRcaller, respectively), as well as on both deletion and insertion subsets (Table 2). The higher genotyping accuracy of deletions over insertions observed with SVJedi-graph is also observed with cuteSV, LRcaller and Sniffles2, at an even more pronounced level, from 3.7 point % difference with cuteSV to 10 with Sniffles2.

SVJedi genotypes a lower proportion of deletions than insertions (85.8% against 93.6%), while all other four genotypers show a relatively stable genotyping rate between both SV types (at most 0.8 point % difference). It is to be noted that cuteSV and LRcaller seem to systematically assign a genotype to all input SVs, thus having a fixed genotyping rate of 100% whatever the SV set.

We also assessed the genotyping performances on the same SV set with two other long-read datasets, one of PacBio CCS (HiFi) technology, and one of ONT technology. The results obtained with the five genotypers are presented in Table 3, along with those previously obtained with the PacBio CLR dataset. SVJedi-graph shows similar genotyping performances for both PacBio datasets, whereas a small decrease in accuracy with ONT reads (of about 4 points %) with a slight increase in rate (of 0.5–1 point %). Contrary to SVJedi-graph, all other genotypers but SVJedi show a higher genotyping accuracy with HiFi and ONT reads compared to CLR reads, Sniffles2 and LRcaller having their best genotyping accuracy on this SV set with the HiFi reads (89.4% and 86.2%, respectively), and cuteSV having its best genotyping accuracy with the ONT reads (92.7%).

**Table 3.** Genotyping accuracy and rate of SVJedi-graph and state of the art genotyping tools on the deletions and insertions of the *High confidence* HG002 SV set, genotyped with PacBio CLR (30×), PacBio CCS (HiFi, 25×), and ONT (40×) reads.

	PacBio CLR		PacBio HiFi		ONT	
Tool	Accuracy (%)	Rate (%)	Accuracy (%)	Rate (%)	Accuracy (%)	Rate (%)
SVJedi-graph	94.6	99.0	94.1	99.5	90.4	100.0
cuteSV	88.7	100.0	91.3	100.0	92.7	100.0
LRcaller	83.6	100.0	86.2	100.0	84.8	100.0
Sniffles2	85.4	99.5	89.4	99.2	88.9	99.8
SVJedi	92.2	90.2	81.3	84.4	90.7	86.2

### 3.4 Applying SVJedi-graph on challenging SVs

As the High confidence set contains only distant SVs, we wanted to explore our method’s performances on a more challenging SV set and we applied SVJedi-graph on another SV set from the HG002 GIAB callsets, called “ClusteredCalls” (that are included in the more difficult Tier 2 regions). This set contains 7003 SV calls that were not included in the High confidence set due to a characterization of lower quality (on breakpoint position and/or genotype). As a matter of fact, 99.5% of these SV calls are within 1 kb of at least one other call. Notably, 58% of deletions overlap at least one other deletion of the set. Additionally, 83% of these SVs fall in regions of Tandem Repeats greater than 100 bp. As the genotypes indicated in the set may not be fully considered as ground truth, we will refer to the genotype quality in terms of % of identical genotypes instead of genotyping accuracy for this set.

All genotyping tools show difficulties to genotype this SV set in comparison to the High confidence SV set, with a decrease of about 20 points of the % of identical genotypes, resulting in around 61–71% of identical genotypes for all tools (Table 4).

**Table 4.** Genotyping rate and % of identical genotypes of SVJedi-graph and state of the art genotyping tools on the deletions and insertions of the *ClusteredCalls* HG002 SV set.^a^

	Global		Deletions		Insertions	
Tool	% of identical genotypes	Rate (%)	% of identical genotypes	Rate (%)	% of identical genotypes	Rate (%)
SVJedi-graph	69.4	81.5	51.5	95.3	80.7	74.9
cuteSV	71.4	100	76.5	100	67.9	100
LRcaller	66.4	100	71.2	100	63.1	100
Sniffles2	61.1	99.7	62.4	100	60.2	99.6
SVJedi	70.3	25.5	47.7	16.9	78.5	31.2

a Genotyping was performed with the 30× CLR PacBio read dataset.

Our tool was able to assign a genotype to 81.5% of these SVs, and 69.4% of them with an identical genotype to the one indicated in the GIAB set, the highest value being 71.4% obtained by CuteSV. SVJedi-graph results on deletions and insertions are very contrasted, both on % of identical genotypes and on genotyping rate. We were able to genotype almost all deletions (95.3%) but with only 51.5% of identical genotypes, while we genotyped less insertions (74.9%) but with the highest % of identical genotypes (80.7%) among the five tools. Both SVJedi-graph and SVJedi showed better performances on insertions than deletions, contrary to the other three SV callers that have in common to rely primarily on read mapping on the reference genome only. SVJedi showed an impaired genotyping rate of 25.5%, which was to be expected considering its difficulties to assign genotypes in the context of close and overlapping SVs.

As concerns SVJedi-graph results, the regions with higher densities of SVs and overlapping SVs did not harbor more missing or different genotypes as in the GIAB set. We could not find any association or relationship between missing and error genotypes with SV size or Tandem Repeat context (as was the case for SVJedi in their publication; Lecompte et al. 2020) to explain the lower concordance of genotypes.

### 3.5 Genotyping a real not curated callset

In addition to the GIAB benchmark SV sets, we tested our method on “raw” SV calling results, which are more likely to contain nested SVs and false positive calls. The idea was to verify that the presence of “noisy” calls (either false positives or poorly described SVs) did not disrupt the genotyping quality of nearby true positive SVs. We applied SVJedi-graph on an SV callset obtained by running a single SV discovery tool, Sniffles (Sedlazeck et al. 2018), on PacBio CLR reads data from HG002 individual, containing 17 637 discovered SVs, with 7921 deletions and 9517 insertions. In this uncurated callset, 13% of the calls are <1 kb apart from another call and 2.3% of the deletions overlap at least one other deletion. SVJedi-graph attributed a genotype to 98% of the 17 637 discovered SVs. To assess the accuracy of these genotypes, we compared these SVs with the ones from the High confidence GIAB HG002 callset, by merging the two sets with Jasmine (Kirsche et al. 2021). Among the 9729 insertions and deletions identified as common between the two sets, 96.4% showed identical genotypes. This is similar and even higher than the accuracy obtained on the curated high confidence set and this indicates that the presence of noise in the SV set does not prevent SVJedi-graph to accurately genotype true positive calls.

Interestingly, common SVs between the two sets did not share exactly the same breakpoint positions, with 57% of them differing by more than 10 bp and 14% by more than 50 bp. This confirms that small imprecision on the breakpoint definition does not impair SVJedi-graph genotyping quality.

### 3.6 Running time and memory usage

The running time and memory requirement of SVJedi-graph and the other genotypers compared on the GIAB HG002 *High confidence* SV set are shown in Table 5. When including the mapping time, SVJedi-graph took less than half an hour to genotype the whole High confidence HG002 SV callset with 30× PacBio CLR reads. It is more than six times faster than all other long-read genotypers (including the mapping time). Notably, the total SVJedi-graph time is similar to the genotyping time alone, for tools that require a mapping to the reference genome (CuteSV, Sniffles2, LRcaller), considering this file may have been obtained previously for other purposes. In terms of memory requirements, SVJedi-graph was in a similar order of magnitude than SVJedi and the two are the less memory demanding tools of the five tested, while LRcaller and Sniffles2 required about 1.5–2 times more memory, and cuteSV about three times more. For LRcaller and Sniffles2, the most memory demanding step among the whole genotyping process was the read mapping with minimap2.

**Table 5.** Running time and memory requirements on the HG002 *High confidence* SV set.^a^

Tool	Running time (min)			Memory (Go)
	Total	Mapping	Genotyping	
SVJedi-graph	29.7	24.8	4.3	19.1
cuteSV	201.9	176	25.9	65.2 (cuteSV)
LRcaller	196.6	176	20.6	29.2 (minimap2)
Sniffles2	233.9	176	57.9	29.2 (minimap2)
SVJedi	189.9	181.9	7.5	13.9

a All tools were run on 20 CPU threads when multi-threading was supported (all but Sniffles2). The total running time shown for SVJedi-graph and SVJedi includes the SV representation step (allelic linear sequences for SVJedi and variation graph for SVJedi-graph) in addition to the mapping and genotyping time. The memory requirement shown for cuteSV, Sniffles2 and LRcaller is the maximum amount of memory used by either the genotyper or minimap2.

For all genotypers, the most time-requiring step is the long-read mapping on the reference genome or on the variation graph for our method. The speed-up of our method is explained by the fact that we chose to use minigraph in its fastest mode, which outputs only alignment coordinates computed over the chaining of minimizers without aligning all bases in between. We also assessed the performances of our method using base-level alignments obtained with minigraph (option “-c”) and another long-read mapper on graph, GraphAligner (Rautiainen and Marschall 2020). Our tests showed that using base level alignments did not improve genotyping rate and accuracy, while drastically increasing the mapping time by at least 15 times.

## 4 Discussion and conclusion

We have presented here the first method and its implementation dedicated to SV genotyping with long reads that is based on a variation graph. The use of a variation graph allows to represent in a single data structure the whole genome along with all described alternative SV alleles. In such a graph, reference and alternative alleles are represented in a strictly equal manner, preventing a potential bias toward the reference allele when mapping reads on it. We have shown on simulated deletion datasets that this approach achieves highly accurate genotyping and the few observed genotyping errors were balanced over both alleles. When applied on a simulated dataset with random inversions, we observed similarly a very high genotyping accuracy without any reference bias (see Supplementary Table S1). Further evidence of the absence of reference bias in SVjedi-graph is the fact that it obtained similar performances between insertions and deletions in the real human benchmark SV set, in contrast to LRcaller and Sniffles.

The second major advantage of using a variation graph is that it allows to represent close and even overlapping SVs efficiently. In particular, for closely located SVs, this representation does not require to choose some haplotypes over all the possible ones. We designed simulated datasets where we controlled the distance or overlap between consecutive simulated SVs in order to precisely assess the impact of such SV distributions on the genotyping performances of the tools. In these simulations, we did not modify the simulated haplotypes and resulting simulated sequencing reads, but we only added additional SVs in the input SV set. The latter are thus to be genotyped as homozygous for the reference allele (0/0). This is the simplest case of close or overlapping variants, since the genotyping signals contained in the reads should remain the same whatever the additional set of SVs. Even in this simplest case, we observed a substantial decrease in genotyping rate or accuracy for all tools except SVJedi-graph as soon as SVs are <500 bp apart or overlapping. It means that in these methods, the quality of the genotyping of a given SV depends on the other SVs present in the SV set, even if absent in the genotyped individual. This was to be expected for SVJedi as it constructs linear allelic sequences around each SV breakpoint independently of the other SVs in the set. As these sequences span up to 5 kb on either side, when the SVs are close, the resulting set of sequences has a lot of redundancy, causing many reads to be filtered out due to their non-unique mapping. This explains why the genotyping rate of SVJedi drops drastically in these results. On the contrary, the stable performances of SVJedi-graph on these datasets demonstrates that the graph-based representation of SVs prevents such non desirable behavior, and allows highly accurate genotyping of clustered and even overlapping SVs.

The real HG002 High confidence benchmark dataset from Genome in a Bottle consortium does not contain such clustered or overlapping SVs, since all SV calls have been selected to be at least 1 kb apart from one another in order to ensure this high confidence in the SV descriptions and genotypes. However, it still contains challenging insertions and deletions, since for instance more than half of them are contained in Tandem Repeat regions greater than 100 bp (Zook et al. 2020, Delage et al. 2020). On this dataset dedicated to the evaluation of SV tools, SVJedi-graph obtained substantial improvement in genotyping accuracy and rate with PacBio CLR and HiFi reads compared to the other tested genotypers and in much less time. Although most other tools had better genotyping performances with ONT reads on this dataset, SVJedi-graph showed a lower genotyping accuracy with respect to the ones obtained with PacBio sequencing reads. This may be explained by the fact that the mapping in SVJedi-graph was performed with the same default parameters of minigraph for all sequencing technologies, whereas the mapping used by other tools was performed with sequencing technology specific parameter presets of minimap2. For the moment, minigraph does not provide parameter presets for the different sequencing technologies, an exploration of mapping parameters that would be best suited to the different technologies could lead to improvements in SVjedi-graph.

On a more challenging SV set with many clustered and overlapping calls, we could have expected based on the simulation results that SVJedi-graph would make an even greater difference with other tools. On the contrary, we obtained poor concordance with the genotypes given as the truth in the HG002 ClusteredCalls set of GIAB, with similar or sometimes worse values than the other genotypers. We investigated numerous factors to explain these results, including the proximity or overlapping of SVs, the genomic context of SVs, the size or genotypes of erroneously genotyped SVs but we did not find any significant association. This absence of relationship with classical factors of errors may argue toward problems of definition of SV breakpoints or inacurrate genotypes in the input SV set. Indeed, the authors of this dataset had deliberately distinguished them from the High confidence set and had warned users that the proximity of the SVs prevented them from being accurately characterized and that they were “potentially complex, compound, or inaccurate” (citation from the repository Readme). The fact that some of the tested genotypers perform better on this particular dataset could be due to biases or errors that are reproducible with similar methods. Indeed, CuteSV and Sniffles genotypings are derived from discovery methods and rely on the same input data, namely reads mapped on the reference genome. They probably use similar read signals that were used to discover these SVs in the first place in GIAB protocols. For instance, they may have used the variation of read depth along the genome to discover some deletions, whereas SVJedi-graph relies exclusively on the breakpoint signals. Notably, those methods performed worse for insertions, for which the signals that can be extracted from mapping to the reference genome are the weakest. In the case of insertions, we notably observed that SVJedi-graph had the best genotyping accuracy but was not able to genotype more than 25% of them due to insufficient read support (less than three reads) for both alleles combined. Interestingly, 85% of these not genotyped insertions are reported as homozygous for the alternative allele in the input set. Such absence of read support even for the reference allele could be explained by inaccuracies in the reported inserted sequence which would be too divergent from the real insertion sequence for reads to map on. These different hypotheses are difficult to settle other than by a manual inspection of each individual case, which would be extremely time-consuming and is outside the scope of this paper.

The uncertainties in this dataset make it therefore poorly suited for precise assessment of tool performances. Conversely, while the High confidence set is an ideal set for benchmarking and comparing tools, it does not reflect the reality of genotyped datasets in practice, which are usually not manually curated and contain more closely located and nested calls, as well as more imprecise and noisy calls. Our experiment on a whole raw and uncurated discovery call set represents a practical and realistic intermediate between the high confidence and the most challenging call sets and showed that SVJedi-graph is usable and obtains good quality results in practice in a few dozens of minutes on a whole human genome dataset.

In conclusion, SVJedi-graph is a fast and efficient tool to genotype SVs with long-read data, that promises to be useful in the ever-growing number of population-scale SV studies.

## Supplementary Material

btad237_Supplementary_Data

## Data Availability

The data underlying this article are available on public repositories whose links are given in the article online supplementary material.
