# Supplementary material for: SVJedi-graph: improving the genotyping of close and overlapping structural variants with long reads using a variation graph
Source: Bioinformatics. 2023 Jun 30;39(Suppl 1):i270–8. doi: 10.1093/bioinformatics/btad237 (PMC10311344; doi:10.1093/bioinformatics/btad237)
Supplement: btad237_Supplementary_Data [file btad237_supplementary_data.pdf]

SVJedi-graph: improving the genotyping of close and overlapping Structural Variants with long reads using a variation graph
Supplementary material

Sandra Romain and Claire Lemaitre

Contents

1 Data accessibility **2**
1.1 Simulated deletion datasets 2
1.2 GIAB analyses 2

2 Tools command lines **2**

3 SVJedi-graph results for inversions **4**

1 Data accessibility

1.1 Simulated deletion datasets

Simulated reads and VCF files used for close and overlapping deletions as well as shifted breakpoint experiments are available for download at `https://data-access.cesgo.org/index.php/s/hAzETo82AUTePFB`

1.2 GIAB analyses

The gold standard call set for individual HG002, provided by Genome in a Bottle (GIAB) Consortium, is available at the following link:

- `ftp://ftp-trace.ncbi.nlm.nih.gov/giab/ftp/data/AshkenazimTrio/analysis/NIST_SVs_Integration_v0.6/HG002_SVs_Tier1_v0.6.vcf.gz`

The High confidence callset was extracted by selecting variants with the tag `PASS` in the FILTER field. The ClusteredCalls callset was extracted by selecting variants with the tag `ClusteredCalls` in the FILTER field.

We used the GRCh37.p13 human genome assembly as the reference genome to genotype both datasets, as it was the one used in the VCF and to identify the SVs.

Sequencing datasets for GIAB HG002

- PacBio CLR: `ftp://ftp-trace.ncbi.nlm.nih.gov/ReferenceSamples/giab/data/AshkenazimTrio/HG002_NA24385_son/PacBio_MtSinai_NIST/`
- PacBio CCS (HiFi): `ftp://ftp-trace.ncbi.nlm.nih.gov/ReferenceSamples/giab/data/AshkenazimTrio/HG002_NA24385_son/PacBio_CCS_15kb/`
- ONT Promethion: `ftp://ftp-trace.ncbi.nlm.nih.gov/ReferenceSamples/giab/data/AshkenazimTrio/HG002_NA24385_son/UCSC_Ultralong_OxfordNanopore_Promethion/`

The PacBio CLR data of the individual HG002 has a sequencing depth of 63x. The sequence data were sub-sampled to a depth of 30x.

2 Tools command lines

SVJedi:

```
#With PacBio CLR and CCS reads
svjedi.py -t 20 -r reference.fa \
    -v sv_set.vcf \
    -i reads.fq

#With ONT reads
svjedi.py -t 20 -r reference.fa \
    -v sv_set.vcf \
    -i reads.fq -d ont
```

SVJedi-graph:

```
svjedi-graph.py -t 20 -r reference.fa \
    -v sv_set.vcf  \
    -q reads.fq
```

Minimap2:

```
#With PacBio CLR reads
minimap2 -ax map-pb -t $t reference.fa reads.fq --MD > minimap2_results.sam
#With PacBio CCS reads
minimap2 -ax asm20 -t $t reference.fa reads.fq --MD > minimap2_results.sam
#With ONT Promethion reads
minimap2 -ax map-ont -t $t reference.fa reads.fq --MD > minimap2_results.sam

samtools view -Sb -q 10 minimap2_results.sam > minimap2_results.bam
samtools sort -o minimap2_results.sorted.bam -O bam minimap2_results.bam
samtools index -b minimap2_results.sorted.bam
```

Sniffles2:

```
#For mapping see Minimap2 commands

#Sniffles2 genotyping
sniffles --input minimap2_results.sorted.bam \
    --genotype-vcf sv_set.vcf \
    --vcf sv_genotype.vcf
```

CuteSV:

```
#For mapping see Minimap2 commands

#CuteSV genotyping with PacBio CLR reads
cuteSV --max_cluster_bias_INS 100 \
    --diff_ratio_merging_INS 0.3 \
    --max_cluster_bias_DEL 200 \
    --diff_ratio_merging_DEL 0.5 \
    --threads $t -Ivcf sv_set.vcf \
    --max_size -1 \
    minimap2_results.sorted.bam reference.fa \
    sv_genotype.vcf ./

#CuteSV genotyping with PacBio CCS reads
cuteSV --max_cluster_bias_INS 1000 \
    --diff_ratio_merging_INS 0.9 \
    --max_cluster_bias_DEL 1000 \
    --diff_ratio_merging_DEL 0.5 \
    --threads $t -Ivcf sv_set.vcf \
    --max_size -1 \
    minimap2_results.sorted.bam reference.fa \
    sv_genotype.vcf ./

#CuteSV genotyping with ONT reads
cuteSV --max_cluster_bias_INS 100 \
    --diff_ratio_merging_INS 0.3 \
    --max_cluster_bias_DEL 100 \
    --diff_ratio_merging_DEL 0.3 \
    --threads $t -Ivcf sv_set.vcf \
```

```
    --max_size -1 \
    minimap2_results.sorted.bam reference.fa \
    sv_genotype.vcf ./
```

LRCaller:

```
#For mapping see Minimap2 commands

#LRcaller genotyping
lrcaller --gtm joint --fa reference.fa \
    minimap2_results.sorted.bam sv_set.vcf \
    sv_genotype.vcf
```

3 SVJedi-graph results for inversions

Inversions

		**SVJedi-graph estimations**			
		0/0	0/1	1/1	./.
**Truth**	0/0	150	0	0	0
	0/1	0	150	0	0
	1/1	0	2	148	0

Genotyping accuracy: 99.6 %

Genotyping rate: 100 %

Supplementary Table 1: Contingency tables of SVJedi-graph genotyping results on a 30x PacBio simulated dataset with 450 randomly simulated inversions. Inversions were randomly simulated on human chromosome 1, by sampling the first breakpoint location in a uniform distribution and choosing the inversion size uniformly between 50 bp and 15 kb. Grey labelled boxes correspond to correct estimation of the genotypes. The number of genotypes that SVJedi-graph fails to assess is indicated by the "./." column.
